# Assessing lead time bias due to mammography screening on estimates of loss in life expectancy

**DOI:** 10.1186/s13058-022-01505-3

**Published:** 2022-02-23

**Authors:** Elisavet Syriopoulou, Alessandro Gasparini, Keith Humphreys, Therese M.-L. Andersson

**Affiliations:** Department of Medical Epidemiology and Biostatistics, Karolinska Institutet, Stockholm, Sweden

**Keywords:** Lead time bias, Loss in life expectancy, Mammography screening, Simulation study

## Abstract

**Background:**

An increasingly popular measure for summarising cancer prognosis is the loss in life expectancy (LLE), i.e. the reduction in life expectancy following a cancer diagnosis. The proportion of life lost (PLL) can also be derived, improving comparability across age groups as LLE is highly age-dependent. LLE and PLL are often used to assess the impact of cancer over the remaining lifespan and across groups (e.g. socioeconomic groups). However, in the presence of screening, it is unclear whether part of the differences across population groups could be attributed to lead time bias. Lead time is the extra time added due to early diagnosis, that is, the time from tumour detection through screening to the time that cancer would have been diagnosed symptomatically. It leads to artificially inflated survival estimates even when there are no real survival improvements.

**Methods:**

In this paper, we used a simulation-based approach to assess the impact of lead time due to mammography screening on the estimation of LLE and PLL in breast cancer patients. A natural history model developed in a Swedish setting was used to simulate the growth of breast cancer tumours and age at symptomatic detection. Then, a screening programme similar to current guidelines in Sweden was imposed, with individuals aged 40–74 invited to participate every second year; different scenarios were considered for screening sensitivity and attendance. To isolate the lead time bias of screening, we assumed that screening does not affect the actual time of death. Finally, estimates of LLE and PLL were obtained in the absence and presence of screening, and their difference was used to derive the lead time bias.

**Results:**

The largest absolute bias for LLE was 0.61 years for a high screening sensitivity scenario and assuming perfect screening attendance. The absolute bias was reduced to 0.46 years when the perfect attendance assumption was relaxed to allow for imperfect attendance across screening visits. Bias was also present for the PLL estimates.

**Conclusions:**

The results of the analysis suggested that lead time bias influences LLE and PLL metrics, thus requiring special consideration when interpreting comparisons across calendar time or population groups.

**Supplementary Information:**

The online version contains supplementary material available at 10.1186/s13058-022-01505-3.

## Background

Several metrics are available to summarise the prognosis of cancer patients. A commonly reported measure is relative survival at specific time points, for example, 5-year relative survival. Under certain assumptions, relative survival can be interpreted as net survival, i.e. as the proportion of patients who would still be alive at a specific time after diagnosis, in a hypothetical world where it is not possible to die from causes other than the cancer of interest [1]. Another measure that is being increasingly used to report cancer survival is the loss in life expectancy (LLE), which is defined as the reduction in life expectancy, following a cancer diagnosis. LLE can be used to quantify the cancer burden for an individual or a whole population and has the advantage that it captures the entire remaining lifespan, rather than being focused on a specific point in time [2]. LLE also has a more intuitive interpretation (compared to relative survival), allowing easier communication to a wider audience including non-specialists [3]. Instead of referring to a hypothetical world of net survival, LLE quantifies the cancer impact in a real-world setting where both cancer and other causes are present. LLE is however strongly age-dependent, as younger patients have more years of life to lose than older patients. An additional measure that improves comparability across ages is the proportion of life lost (PLL), calculated by adjusting LLE with the expected remaining lifespan.

Various studies have evaluated how LLE, after a cancer diagnosis, varies over calendar time and across population groups [4–6]. Such comparisons allow the exploration of temporal changes in cancer care as well as the identification of especially affected groups. Typically, for screened cancers, LLE estimates are assumed to not be affected by lead time bias or no mention of the possibility is made. This is in contrast to relative survival estimates where researchers usually recognise the possibility. To our knowledge, no study has formally assessed the impact of lead time on LLE. Lead time is the time between the time of diagnosis of cancer via screening and the time that cancer would have been diagnosed in the absence of screening (symptomatically) [7–9]. Earlier detection of tumours results in prolonged survival times even when there are no actual improvements in time to death. Screening can affect survival times both through real improvements in survival (e.g. due to tumours being diagnosed at earlier stages which leads to better treatment options and better chances of cure or prolonging of life) as well as artificially (adding lead time).

Differences in LLE across socioeconomic or education groups have been evaluated in previous studies [10, 11]. Although many factors have been suggested as potential drivers for the observed differences in LLE across such groups, further research is required to improve understanding of how specific differences arise [12–17]. For cancers that are screened (e.g. breast, prostate, colorectal) lead time bias is a potential contributing factor. The uptake of screening has been found to vary across socioeconomic groups, even in countries where screening programmes are available on a national level, with individuals from lower socioeconomic groups being less likely to attend screening [18–21]. Thus, lead time bias may be larger among individuals from higher socioeconomic groups. Of course early detection can have real as well as artificial advantages; partitioning the effect of screening into real and artificial survival improvements is though challenging as it requires knowledge of what would have happened in the absence of screening.

A previous study assessed the impact of lead time bias on relative survival estimates through a simulation-based approach based on Swedish breast cancer data [22]. The analysis showed that in some settings the bias on relative survival estimates reached 4.0–5.7 percentage points and the authors concluded that lead time bias should not be neglected when interpreting trends in breast cancer survival or differences between population groups in settings where there could be differences in screening participation. In this paper, we use a similar simulation-based approach to assess the impact of lead time bias on LLE and PLL metrics and carry out a sensitivity analysis, assuming different screening programmes of low, moderate and high screening sensitivity and allowing for different attendance rates across screening visits. We compare estimates of marginal 10-year relative survival, LLE and PLL in the absence of screening with equivalent estimates in the presence of a screening programme, in order to calculate the lead time bias.

The remainder of the paper is structured as follows. First, we describe the relative survival framework and introduce the metrics of interest, i.e. loss in life expectancy and proportion of life lost. In addition to conditional measures, we also define marginal measures and show how to obtain estimates using regression standardisation. Following, we describe the simulation-based approach performed to assess bias in LLE and PLL estimates due to lead time as well as the findings of the simulation study. Finally, we summarise the main findings and provide a discussion on the strengths and limitations of our approach.

## Methods

### Excess mortality and relative survival

When analysing cancer registry data the event of interest is usually death due to a specific cancer. However, competing events, such as death due to other causes, may be present. Cancer prognosis is often summarised as relative survival (and its mortality analogue, excess mortality) [1]. The popularity of the relative survival metric is driven by the fact that it circumvents issues regarding the availability and the accuracy of the cause of death information (that can be challenging in a cause-specific approach) and provides estimates without relying on the cause of death classification [23]. The excess mortality approach accounts for non-cancer mortality by incorporating the expected mortality rates of a comparable group which is assumed to be free from the cancer under study. Individuals from cancer and non-cancer populations are assumed to have similar characteristics such as age, sex and calendar year.

Using the relative survival framework, the all-cause mortality rate of an individual *i* with covariate pattern $\varvec{Z=z_{i}}$ can be written as:

1 $$\begin{aligned} h(t|\varvec{Z=z_i}) = h^*(t|\varvec{Z_1=z_{1i}}) + \lambda (t|\varvec{Z_2=z_{2i}}) \end{aligned}$$

where $h^*(t)$ is the expected mortality rate and $\lambda (t)$ is the excess cancer mortality rate. The expected mortality rates are considered to be known and are obtained from stratified population lifetables of the general population. In Eq. (1), $\varvec{Z}$ denotes the set of all covariates of interest which can be partitioned into two subsets: $\varvec{Z_1}$ and $\varvec{Z_2}$ denoting the covariates for which there is variation in expected and excess mortality, respectively. Covariates in $\varvec{Z_1}$ correspond to the covariates for which the population lifetables are stratified. Often $\varvec{Z_1}$ will be a subset of $\varvec{Z_2}$: in that case $\varvec{Z_2}$ and $\varvec{Z}$ will be the same.

By transforming to the survival scale, the all-cause survival of the $i\text {th}$ individual is given by

2 $$\begin{aligned} S(t|\varvec{Z=z_i}) = S^*(t|\varvec{Z_1=z_{1i}}) R(t|\varvec{Z_2=z_{2i}}) \end{aligned}$$

where $S^*(t|\varvec{Z_1=z_{1i}})$ and $R(t|\varvec{Z_2=z_{2i}})$ denote the expected and relative survival probability, respectively. Once again, the expected survival probability is considered to be known/fixed and is obtained from available population lifetables of a comparable population.

Under assumptions, relative survival is interpreted as survival in a hypothetical world where the cancer of interest is the only possible cause of death: (1) the two competing events are conditionally independent and (2) the population lifetables are sufficiently stratified [24].

### Loss in life expectancy

Cancer prognosis can also be quantified in terms of loss in life expectancy. The loss in the life expectancy (LLE) for a cancer patient is defined as the difference between the life expectancy of an individual with similar characteristics in the general population that is free of the cancer of interest and the life expectancy of that patient. This can be written as:

$$\begin{aligned} \text {LLE}(\varvec{Z}=\varvec{z_{i}})=\int _{0}^{t_{\max }}S^*(t|\varvec{Z_1}=\varvec{z_{1i}})\hbox {d}t \\ -\int _{0}^{t_{\max }}S(t|\varvec{Z}=\varvec{z_i})\hbox {d}t \end{aligned}$$

In theory, the integrals should have limits of 0 and $\infty$. In practice, a time point $t_{\text {max}}$, denoting an assumed time at which survival functions become zero, is used for the upper limits. However, the survival curves needed for the calculation of LLE are usually not observed up until $t_{\text {max}}$, due to limited follow-up, and therefore they have to be estimated by extrapolating beyond available data. Andersson et al. [2] showed how to consistently extrapolate the survival curves using flexible parametric models. The main idea is to replace the all-cause survival using Eq. (2) and to extrapolate the relative and expected survival curves instead of the all-cause survival:

$$\begin{aligned} \widehat{\text {LLE}}(\varvec{Z}&=\varvec{z_{i}})= \int _{0}^{t_{\max }}S^*(t|\varvec{Z_1}=\varvec{z_{1i}})\hbox {d}t \\ &-\int _{0}^{t_{\max }}S^*(t|\varvec{Z_1}=\varvec{z_{1i}})\times {\widehat{R}}(t|\varvec{Z_2}=\varvec{z_{2i}})\hbox {d}t \end{aligned}$$

While LLE is a very relevant and easily interpreted metric, it is however highly dependent on age since younger individuals have more years of life to lose. To improve comparability across ages, a proportional measure can be obtained in addition to the absolute measure. The proportion of life lost (PLL) for a cancer patient *i* with covariate vector $\varvec{Z}=\varvec{z_i}$ is equal to their loss in life expectancy divided by the life expectancy of an individual with similar characteristics ($\varvec{Z_1}=\varvec{z_{1i}}$) from the general population:

$$\begin{aligned} \text {PLL}(\varvec{Z}=\varvec{z_i})=\frac{\text {LLE}(\varvec{Z}=\varvec{z_{i}})}{\int _{0}^{t_{\max }}S^*(t|\varvec{Z_1}=\varvec{z_{1i}})\hbox {d}t} \end{aligned}$$

By using the expected life expectancy of an individual in the denominator, PLL accounts for higher life expectancy among younger individuals and varies less across ages in comparison to LLE. However, PLL is a relative measure and does not allow conclusions about whether the loss in life expectancy is meaningful or not in practice. Absolute measures, such as the LLE, are better for understanding whether the impact of cancer is clinically meaningful for an individual or a population. Thus, we encourage the estimation of both LLE and PLL as each measure can help us understand different aspects of the cancer impact.

### Marginal measures

In addition to conditional measures, marginal measures over the whole population can also be defined. Marginal estimates have a simple interpretation as a single measure for each time point of interest, even after fitting complex models with nonlinear effects and interactions between covariates. There are many ways to obtain marginal estimates, but in this paper, the focus is on regression standardisation methods [25]. Standardised estimates are obtained by averaging over the marginal distribution of some covariates. For instance, the marginal 10-year relative survival can be estimated by the standardised relative survival [26]:

3 $$\begin{aligned} \frac{1}{N} \sum _{i=1}^N {\widehat{R}}(t=10|\varvec{Z_2} = \varvec{z_{2i}}) \end{aligned}$$

where *N* is the number of individuals in the study population and ${\widehat{R}}(t=10|\varvec{Z_2} = \varvec{z_{2i}})$ is the predicted 10-year relative survival for individual *i*. Equation (3) is an average over the individual predictions of everyone in the study population.

Quite often a natural choice for the standard covariate distribution is the observed sample distribution, as described in Eq. (3). However, depending on the research question, an external standard distribution might be more appropriate in some settings, e.g. when we want to apply a reference age distribution in two contrasting populations such as countries [27]. For instance, the marginal 10-year relative survival can be derived as the externally age-standardised relative survival [26]:

4 $$\begin{aligned} \frac{1}{N} \sum _{i=1}^N w_i \times {\widehat{R}}(t=10|\varvec{Z_2} = \varvec{z_{2i}}) \end{aligned}$$

where $w_i$ denotes the weight for individual *i*. Weights $w_i$ are calculated as the ratio of the proportion within an age group in the reference population ($w_i^s$) to the proportion of the corresponding group in the study population ($a_i$) and can be written as $w_i = w_i^s / a_i$. Weights above one are applied to groups that are underrepresented in the study population compared with the standard population. Similarly the marginal LLE can be estimated as the externally age-standardised LLE:

5 $$\begin{aligned} \frac{1}{N} \sum _{i=1}^N w_i \times \widehat{\text {LLE}}(\varvec{Z} = \varvec{z_i}) \end{aligned}$$

and the marginal PLL by the externally age-standardised PLL:

6 $$\begin{aligned} \frac{1}{N} \sum _{i=1}^N w_i \times \widehat{\text {PLL}}(\varvec{Z} = \varvec{z_i}). \end{aligned}$$

### Simulation approach

To assess the impact of lead time on estimates of LLE and PLL, we applied a simulation-based approach similar to the one described by Andersson et al. [22]. The study was coded using R and Stata, and all simulation code is openly available online at https://github.com/syriop-elisa/lead_time_bias. We first simulated data in the absence of a screening programme. We generated birth cohorts consisting of 10,000 individuals for every year between 1870 and 1965. This corresponds to approximately one fifth of the actual size of birth cohorts of females in Sweden. The potential onset of breast cancer tumour was simulated based on age-specific probabilities of tumour onset (see details in “Appendix”). For simplicity, only one breast cancer was allowed for each individual. For individuals with onset of breast cancer, we also simulated their tumour growth, tumour detection and age at death (see details in “Appendix”). These correspond to a scenario when there is no screening programme applicable and only symptomatically diagnosed tumours are considered.

After simulating data without screening, we then imposed different screening scenarios on the same simulated data. Under screening, some individuals have the same symptomatic diagnosis as in the no screening scenario, some individuals are diagnosed earlier and some individuals that died before being detected symptomatically in the no screening scenario are diagnosed through screening and included in the analysis. We considered a range of screening scenarios, assuming different screening sensitivities and attendance probabilities.

For our analysis, we first estimated marginal 10-year relative survival, LLE and PLL from the simulated data in the absence of screening (only symptomatic cases). We then obtained estimates of marginal 10-year relative survival, LLE and PLL after imposing different screening scenarios. We compared estimates when no screening is imposed to estimates in the presence of a screening programme to obtain the lead time bias. To allow calculating the lead time, the actual time of death was not changed for screen-detected cases. In practice, screening might also result in improved survival outcomes of patients but the aim of our simulation study was to isolate the impact of lead time bias. We repeated the analysis to create 200 simulated datasets.

More details on simulating time to death and screening scenarios as well as obtaining the estimates of interest and lead time bias can be found in the following sections.

#### Time to death

For each individual, we generated both a time to death from cancer and a time to death due to other causes; the minimum between the two was taken as the time to death.

Time to death due to cancer was measured from age at symptomatic diagnosis, and simulated from a flexible parametric relative survival model. Flexible parametric models (FPM) are based on a generalisation of the Weibull distribution and explicitly estimate the baseline log-cumulative hazard using restricted cubic splines for the logarithm of time rather than assuming linearity with time [28, 29]. In this way, FPMs allow a wide range of hazard functions to be captured. The choice for the number of knots used to create the spline function (or number of degrees of freedom, df, which is equal to the number of knots minus one) is dictated by the complexity of the available data and is made by the analyst. The relative survival FPM used to generate time to death due to cancer in the simulation included age at symptomatic diagnosis as a continuous variable and assumed 3 degrees of freedom for the baseline excess hazard. The fitted model can be written mathematically as

7 $$\begin{aligned} \ln (\Lambda (t)) = s(\ln (t)|\varvec{\gamma }, \varvec{k_0}) + \alpha \text {Age} \end{aligned}$$

where $\ln (\Lambda (t))$ is the log cumulative excess hazard, $s(\ln (t)|\varvec{\gamma }, \varvec{k_0})$ is a restricted cubic spline function of log time with $\varvec{\gamma }$ spline coefficients and $\varvec{k_0}$ knots for the log baseline cumulative hazard, and $\alpha$ is the regression coefficient for age at symptomatic diagnosis. The parameters values were obtained by fitting the model to real data from the Swedish Cancer Registry on breast cancers diagnosed in Sweden between 1970 to 1974 and can be found at https://github.com/syriop-elisa/lead_time_bias. For individuals with cancer onset, age at death from breast cancer was calculated as the summation of their age at symptomatic detection and the survival time from breast cancer.

For the above, we used an old dataset on breast cancers in Sweden and survival was lower than breast cancer survival estimates reported using more recent data; LLE was also higher. In particular, 10-year relative survival was 50.7%, LLE was equal to 7.74 years, and the PLL was 42.6%. These are externally standardised estimates using the age distribution of a reference population [27]. We chose to include years prior to the introduction of mammography screening in Sweden as it was important to use incidence rates that are not affected by screening. The worse prognosis in our data should only have a small impact on bias estimates.

Time to death due to causes other than breast cancer was simulated from birth. This was generated from exponential distributions using mortality rates in the Swedish population life tables stratified by sex, age and calendar year and assuming 100 years for the longest possible lifetime. Since our study population consists of women, only a subset of the life tables is utilised (i.e. the expected mortality rates of women). Specifically, we used the inversion method (as described by Bender et al. [30]):

$$\begin{aligned} T= - \frac{\log (U)}{\text {mortality rate}} \end{aligned}$$

where *U* is a random variable with $U \sim U(0,1)$. More specifically, expected mortality rates for years 1870–2011 were obtained from the Human Mortality database [31], while mortality rates beyond year 2011 were obtained from mortality projections created by Statistics Sweden [32]. To account for the increase in attained age, we applied a different rate for each year of follow up. If a value greater than one was generated, the individual was assumed to be alive at the start of the next year-long interval; otherwise, if the value was less than one, it was assumed that the individual had died in the interval.

Finally, for each individual, age at death was defined as the minimum of age at death due to breast cancer and age to death due to other causes.

#### Screening sensitivity and attendance scenarios

We also imposed a mammography screening programme where individuals were assumed to be invited to screening every second year from the age of 40 until the age of 74. In this setting, some individuals were diagnosed earlier, via screening, while others were diagnosed symptomatically. Under screening there will also be some tumours detected that would have remained undetected in the absence of screening (because the individuals would have died due to causes different to cancer before they are detected symptomatically). Screening sensitivity was assumed to follow the logistic function:

$$\begin{aligned} \text {Screening sensitivity} = \frac{\exp (\beta _1 + \beta _2d)}{1+\exp (\beta _1 + \beta _2d)} \end{aligned}$$

where *d* denotes the tumour diameter at the relevant point in time. As in Andersson et al. [22], values $\beta _1$ and $\beta _2$ were selected based on the previous work of Abrahamsson and Humphreys [33]. In particular, Abrahamsson and Humphreys estimated parameter values for screening sensitivity by fitting the growth model (described in “Modelling tumour growth and symptomatic detection” section of the “Appendix”) to data on tumour size and screening history from Swedish women with postmenopausal breast cancer [34]. In their approach, the authors allowed screening sensitivity to depend on both mammographic density and tumour size. Andersson et al. used the point estimate as well as the upper and lower confidence limits of the parameters (obtained by Abrahamsson and Humphreys [33]) to simulate scenarios of moderate ($\beta _1=-\,5.04$ and $\beta _2=0.56$), low ($\beta _1=-\,5.45$ and $\beta _2=0.48$) and high ($\beta _1=-\,4.67$ and $\beta _2=0.65$) screening sensitivity, respectively, assuming the same mammographic density for all individuals [22]. The values of $\beta _1$ and $\beta _2$ result in screening sensitivities of 0.58, 0.84 and 0.96 for low, moderate and high sensitivities, respectively, for a tumour that is 12 mm wide. We use the same values/criteria here.

In addition to considering three screening sensitivities, we also considered two screening attendance scenarios. We allowed some individual to have a higher probability to attend their screening and some individuals to miss some visits but attend others. Specifically, attendance at each screening visit was assumed to be either *perfect*, where everyone attends all screening visits, or *imperfect*, where 80% of the individuals attend each scheduled screen visit with a probability of 0.9 and 20% of individuals attend each scheduled screen visit with a probability of 0.15.

#### Estimates of interest

For each simulated dataset, we obtained externally age-standardised estimates of 10-year relative survival, LLE and PLL for individuals diagnosed during the years 1970–1974, both in the presence and in the absence of screening. For this, we fitted a relative survival FPM with 3 degrees of freedom for the baseline excess hazard, including age as a continuous, linear variable while allowing for a time-dependent effect (3 degrees of freedom). Follow-up was assumed to occur from time of diagnosis until date of death or 12 years after diagnosis, whichever occurred first (mimicking administrative censoring). After fitting the model, estimates were obtained by averaging the individual specific predictions and utilising external weights as in Eqs. (4), (5) and (6). We used the international cancer survival standards (ICSS) weights to match the age distribution of a reference population [27]. The weights correspond to an age distribution with proportions 7%, 12%, 23%, 29% and 29% for age groups 44 and below, 45–54, 55–64, 65–74 and 75 and above, respectively. These are standard weights that are often used to conduct comparisons between population groups and countries that may have different age distribution.

#### Lead time bias

To be able to isolate the artificial changes in our estimates due to lead time the actual survival time was not changed for the screen-detected cases and remained the same as in the no screening scenario. Thus, estimates under no screening correspond to the *actual* values. For each of the three screening sensitivity scenarios and each of the two attendance scenarios, we calculated the difference in estimates compared to the no screening setting. By averaging the differences across 200 simulations, we obtained the lead time bias (on the absolute scale). The relative bias was also calculated by dividing lead time bias with the point estimates obtained under no screening.

## Results

As shown in Andersson et al. [22], the simulation strategy resulted in data that correspond well with registry data from the Swedish Cancer Registry and the Stockholm-Gotland regional quality register for breast cancer, with slightly older age distribution and slightly larger tumours in the simulation datasets compared to data from the register. More specifically, in the absence of screening there were 2955 cases diagnosed on average for each simulated dataset; this was slightly higher in the presence of screening (Table 1). Age at diagnosis was lower under screening settings, with a median of 60 years for screening assuming moderate screening attendance and imperfect attendance and 61 under no screening. There were also more tumours with a smaller size among the screening setting. More details on the simulated data in the presence of screening assuming moderate screening sensitivity and imperfect adherence as well as the simulated data in the absence of screening can be found as averages over 200 simulations in Table 1.

**Table 1:** Desciptives (averages from 200 simulations) for the simulated datasets without screening and with screening assuming moderate screening sensitivity and imperfect attendance

	No screening	Screening
Number diagnosed	2955	3010
Mean age at diagnosis	61	60
25th percentile of age	50	49
Median age	62	61
75th percentile of age	73	72
% Dead within 12 years	63.0	60.9
% Size smaller than 17.5	39.0	56.8
% Size 17.5–32.5	44.2	30.2
% Size 32.5–47.5	12.2	8.9
% Size larger than 47.5	4.6	4.1

The proportion of screen-detected tumours increased from 35.2 to 53% for low to high screening sensitivity scenarios when perfect screening attendance was assumed for all individuals (Table 2). These values are averages across 200 simulated datasets. When an imperfect attendance was allowed, with some individuals missing some visits but attending others, the proportion of screen-detected tumours ranged from 27.1 to 42.1% for low to high screening sensitivity scenarios. The screen-detected proportions in our simulated data were much higher among ages 40–74 when individuals were invited for screening, ranging from 38.4 to 58% across screening sensitivity scenarios and assuming imperfect attendance (see Additional file 1: Table S1). These proportions are in good agreement with reports from countries with mammography screening programmes available on a national level: approximately half of all cases of breast cancer in Sweden are detected during mammography screening [35], while the screen-detected proportion in the UK between April 2008 and March 2009 was 27% [36]. Mean and median lead time among screen-detected tumours were 2.42 and 1.32 years, respectively, for a moderate screening sensitivity scenario and allowing for imperfect attendance (Table 2). These values suggest that many of the screen-detected tumours would have been detected symptomatically within the first two years following their screening detection. The mean and median lead time were slightly lower for a low sensitivity screening programme and slightly higher for a high sensitivity screening programme, with negligible differences between the perfect and imperfect screening attendance scenarios.

**Table 2:** Proportion screen detected, and mean and median lead-time (in years) among screen detected cases in different simulation screening scenarios

Attendance	Screening	Number diagnosed	% screen detected	Lead time (mean)	Lead time (median)
Perfect	Low	2999 (2901–3098)	35.2 (33.7–37.1)	2.01 (1.83–2.22)	1.01 (0.92–1.10)
Perfect	Moderate	3028 (2925–3136)	45.1 (43.2–47.1)	2.45 (2.27–2.64)	1.34 (1.22–1.45)
Perfect	High	3062 (2959–3171)	53.0 (51.0–54.9)	2.98 (2.80–3.22)	1.72 (1.61–1.84)
Imperfect	Low	2988 (2887–3075)	27.1 (25.1–28.8)	1.98 (1.74–2.27)	1.01 (0.91–1.11)
Imperfect	Moderate	3010 (2904–3106)	35.3 (33.7–36.8)	2.42 (2.22–2.67)	1.32 (1.19–1.45)
Imperfect	High	3035 (2928–3143)	42.1 (40.6–44.0)	2.93 (2.71–3.19)	1.70 (1.55–1.83)

All numbers are averages (with 2.5 and 97.5 percentiles in parenthesis) based on 200 simulations

**Table 3:** Estimates of externally age-standardised 10-year relative survival (RS) in percentages, loss in life expectancy (LLE) in years and proportion of life lost (PLL) in percentages in the absence of screening as well as in the presence of screening across different screening sensitivities and attendance scenarios

Attendance	Screening	10-Year RS	LLE	PLL
—	None	50.96 (48.18–54.04)	8.08 (7.62–8.50)	44.13 (41.58–46.39)
Perfect	Low	52.35 (49.36–55.44)	7.80 (7.37–8.20)	42.95 (40.56–45.13)
Perfect	Moderate	53.47 (50.72–55.91)	7.63 (7.24–8.00)	42.18 (39.95–44.21)
Perfect	High	54.81 (52.27–57.50)	7.48 (7.08–7.89)	41.47 (39.29–43.73)
Imperfect	Low	52.05 (49.29–54.91)	7.87 (7.45–8.29)	43.22 (40.84–45.56)
Imperfect	Moderate	52.83 (49.69–55.65)	7.74 (7.33–8.16)	42.69 (40.37–44.95)
Imperfect	High	53.83 (51.24–56.47)	7.63 (7.20–8.07)	42.16 (39.88–44.65)

All numbers are averages (with 2.5 and 97.5 percentiles in parenthesis) based on 200 simulations

**Fig. 1:**
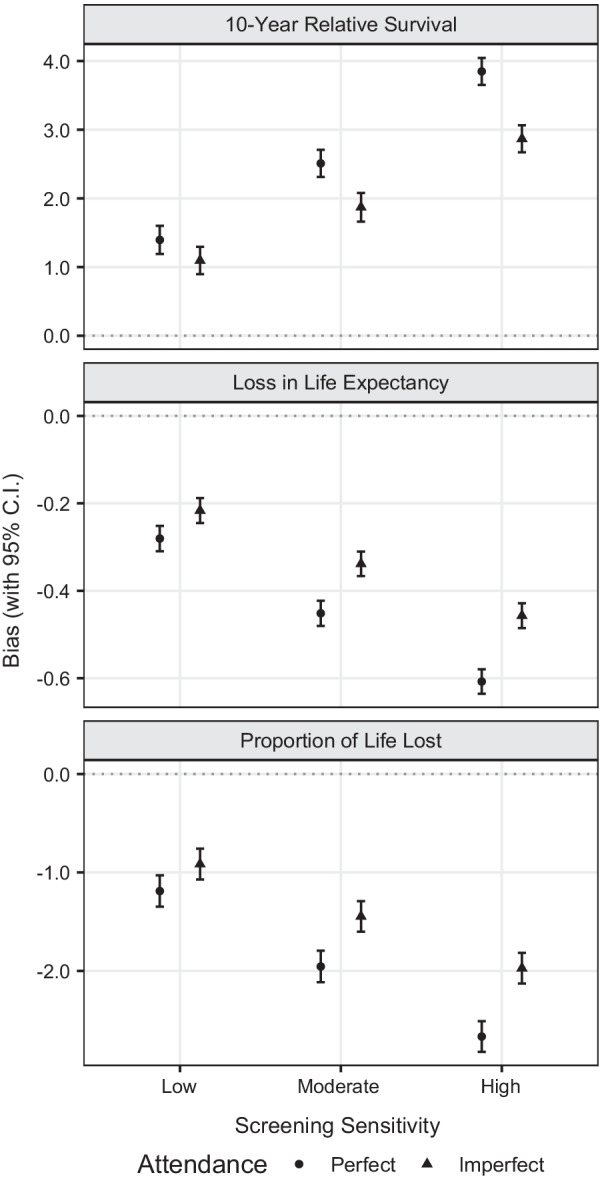
Bias for externally age-standardised 10-year relative survival, loss in life expectancy (LLE) and proportion of life lost (PLL) across different screening sensitivities and attendance scenarios, with 95% confidence intervals based on the Monte Carlo error for bias (across 200 simulations). Bias was obtained as the difference to the setting in which no screening is imposed and all cases are symptomatic

In the absence of screening, by averaging across 200 simulated datasets, externally age-standardised 10-year relative survival was 50.96%, LLE was 8.08 years and PLL was 44% (Table 3). As expected due to the artificially prolonged survival times, when a screening programme was imposed, estimates of 10-year relative survival were higher while estimates of LLE and PLL were lower. When comparing estimates in the absence of screening to those obtained in the presence of screening, a maximum absolute bias of approximately 4 percentage points was observed for 10-year relative survival under screening with high sensitivity and perfect attendance (Fig. 1). The bias was reduced with lower screening sensitivity and was also lower under imperfect screening attendance but it remained higher than one percentage point across all scenarios. A similar pattern was also observed for the bias of LLE and PLL. The bias of LLE was negative with the absolute bias varying from 0.3 to 0.6 years across low, moderate and high screening sensitivities when perfect attendance was assumed. When imperfect attendance was allowed it was reduced but it remained above 0.2 years. Negative bias was also observed for PLL with the absolute bias varying from approximately 1–3 percentage points across all scenarios. The confidence intervals for the bias, which were calculated based on Monte Carlo errors, were narrow for all metrics of interest (Fig. 1).

**Fig. 2:**
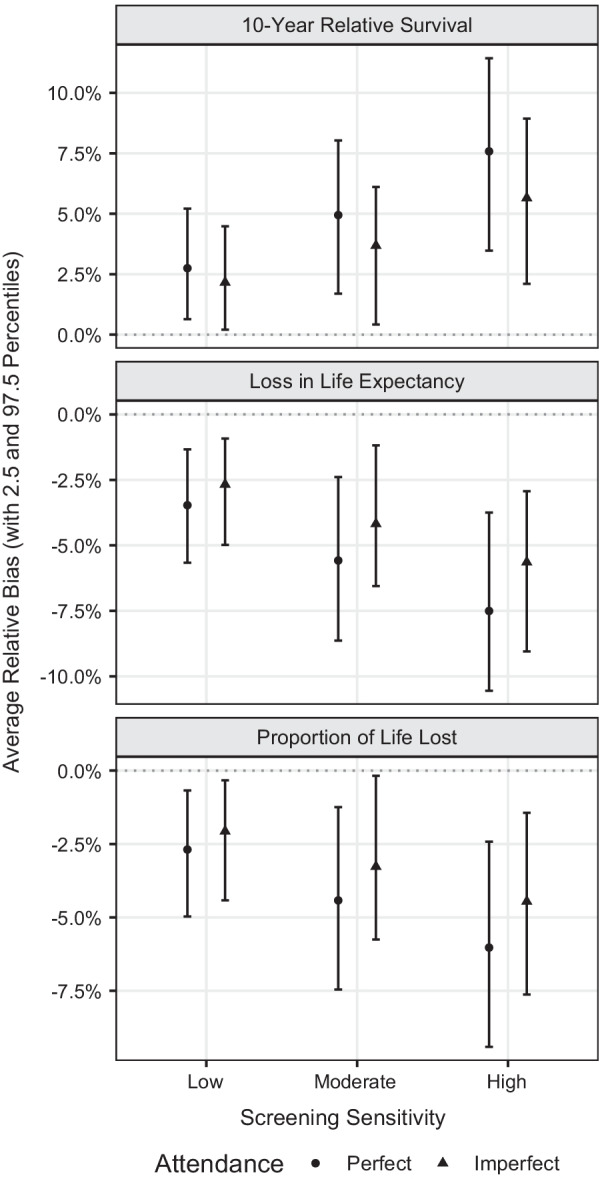
Average relative bias for externally age-standardised 10-year relative survival, loss in life expectancy (LLE) and proportion of life lost (PLL) across different screening sensitivities and attendance scenarios, with 2.5 and 97.5 percentiles based on 200 simulations. The reference scenario is the setting in which no screening is imposed and all cases are symptomatic

Figure 2 shows the average relative bias for each screening sensitivity and screening attendance scenario, with the 2.5 and 97.5 percentiles across 200 simulations. The average relative bias was of similar size for 10-year relative survival and LLE but of opposite directions. For instance, when a high sensitivity screening programme was imposed and perfect attendance was assumed, the estimates of 10-year relative survival were overestimated by 7.6%. However, under the same screening scenario, estimates of LLE were underestimated by 7.5%. Slightly lower relative bias was observed for PLL. In general, lower relative bias was observed for lower screening sensitivity and imperfect screening attendance. More detailed information on the actual values of bias, both on the absolute and relative scale, can be found in the supplementary material (see Additional file 1: Tables S2 and S3).

## Discussion

We have assessed the impact of lead time bias, which, for breast cancer, is introduced in the presence of mammography screening, on the estimation of loss in life expectancy metrics using a simulation-based approach. Different scenarios were assumed for screening sensitivity as well as screening attendance to allow for settings where individuals may attend some visits but miss others. Estimates of LLE and PLL in the absence of screening were compared with estimates when screening was imposed to obtain the lead time bias. Earlier detection through screening can result in both real and artificial advantages. However, partitioning the effect of screening into real and artificial survival improvements is challenging as it requires knowledge of what would have happened both in the presence and in the absence of screening. In our simulation-based approach stage shifting due to screening was not allowed as the aim was to isolate the impact of lead time in estimates of LLE and PLL and not to look at improvements in survival due to screening. Estimates in the absence of screening correspond to the actual values, while estimates in the presence of screening are influenced by prolonged survival times due to an earlier diagnosis even though the time of death remains unchanged under screening in our simulation. The largest absolute bias for LLE was 0.61 years for a high screening sensitivity scenario and assuming perfect attendance. The absolute bias was reduced to 0.46 years when the perfect attendance assumption was relaxed to allow for imperfect attendance across screening visits. Bias was also present in the estimates of the PLL metric.

Bias for LLE remained above 0.2 years across all scenarios, suggesting that, even in situations when there are no real improvements in survival, loss in life expectancy metrics may be influenced by lead time. Consequently, lead time bias might explain part of the differences in LLE that have been previously reported across population groups (such as socioeconomic groups). For instance, a recent Swedish study investigated differences in LLE by education groups and found that women belonging to a lower education group and diagnosed with breast cancer at age 55 lose on average 5.42 years due to cancer, while women diagnosed at the same age but belonging to a high education group, lose on average 5.03 years, resulting in a difference of 0.39 years [10]. Differences varied across ages but the gap between different education groups persisted. Another study using English registry data found a difference of 0.62 years in marginal LLE between individuals from the least and most deprived groups, with the most deprived groups losing the most years of life [11]. Even though the drivers for participation in mammography screening are still not well-understood, potential factors of non-attendance such as low socioeconomic status or low education have been reported before [37–39]. As we showed in our simulation approach, if screening attendance is lower in some groups, differences in prognosis across population groups could partly be explained by lead time bias that introduces artificial improvements in survival. In a real-world setting, the reasons for the observed differences across population groups reported by several studies are likely multifactorial, including both cancer-related and other factors. In addition to lead time bias, earlier diagnosis through screening may also result in better treatment options and thus better prognosis for some subgroups. Another factor that may also explain part of the differences is pre-existing comorbidities that circumvent some groups from receiving treatment [40].

It is important to recognise that our analyses are based on assumptions about the natural history of breast cancer. The tumour growth rates used for the simulation are based on previously published research using Swedish data. The average rate is similar to what has been estimated from in vivo studies; slightly slower than [41], but slightly faster than [42, 43], although one of these in vivo studies [43] was based on ER+ breast cancers only, which are known to grow more slowly than ER– cancers/cancers on average. If our rates are too fast then we will have underestimated the true lead time, and vice-versa. With slower growth rates there would be more screen-detected tumours. Furthermore, a possible bias introduced by screening is overdiagnosis. Overdiagnosis is present in our simulation, in one sense, as indicated by the small differences in the number diagnosed between the screening and no screening scenarios (Table 1). Often overdiagnosis corresponds to the detection of tumours that would have remained undetected in an individuals lifetime if they had not attended screening [44]. This will to some extent always be present when cancers are diagnosed earlier. However, overdiagnosis is particularly an issue for slow-growing or even regressive tumours, and in situ cancers. The tumour growth model applied to generate the data in our simulation assumes that all tumours will eventually show symptoms and was based on an only invasive breast cancer, so we did not consider overdiagnosis due to indolent or in situ cancer. In situ tumours are often excluded in analyses of cancer registry data [10, 11] and so our simulation-based approach provides a good realisation of such analyses.

Even though we attempted to imposed a screening programme very similar to the current one in Sweden (women aged 40–74 invited every second year), screening settings have changed over time and in different counties within Sweden. Furthermore, our simulation was based on data in Sweden from the early 70s before the introduction of mammography screening, and there have been many improvements in survival since then. However, for this project, it was particularly important to use incidence and survival rates that are not affected by screening. Even though these rates may differ from today’s rates, the lead time bias observed in our simulations is also relevant to recent data. We note also that Andersson et al. [22], considered scenarios with lower and higher survival than the one considered in our simulation and found similar bias across all survival scenarios (except for differences due to random variation). Finally, even though our approach is developed for breast cancer data, it could be modified to other cancer types that might be affected by lead time bias. These may even include cancers for which there is no screening programme, but which are diagnosed earlier in the natural history of the disease for some groups compared to others (or across calendar time).

In this paper, we focus on estimating LLE using age at cancer diagnosis as the starting point, which is a popular approach for estimating the impact of a cancer diagnosis on the remaining lifespan [4–6, 10, 11]. In this way, the life expectancy of a cancer patient at the age of diagnosis is compared to their expected life expectancy had they not had a cancer diagnosis (at that age). As age of diagnosis will differ in the presence and absence of screening, we showed that LLE is subject to lead time bias. An important feature of the LLE is that by comparing the life expectancy of the cancer patients to that of the general population, LLE captures the years lost due to cancer both directly (e.g. failure of a vital organ in which the tumour developed) and indirectly (e.g. adverse treatment effects). A related measure that might be less influenced by lead time is given by the number of life years lost, which can be estimated using age at death among the patients who died from the cancer of interest as the starting point [45]. However, this corresponds to a different quantity compared to our definition of LLE. Under this approach, the number of years lost for a cancer patient is calculated based on the expected remaining life expectancy at their age at death from cancer. Thus, only the years lost directly due to cancer are estimated. Moreover, as this measure requires to be able to identify patients who died from their cancer, accurate cause of death information is required. This may not be readily available, or may be problematic in some cases. Finally, all patients who died from cancer are included in the calculation of this second measure, regardless of when they were diagnosed, making it inappropriate for drawing conclusions for a specific cohort of patients, e.g. life years lost among patients diagnosed in a specific calendar year.

## Conclusions

We have shown that lead time bias introduced due to mammography screening may result in seemingly improved estimates of LLE and PLL even when there is no real improvement. It is therefore important to carefully consider the impact of lead time bias when comparing life expectancy measures across time or across population groups of cancer patients.

### Supplementary Information

**Additional file 1.** Supplementary Tables S1, S2 and S3.

## Data Availability

The simulation-based approach described in this paper was coded using R and Stata, and all simulation code is openly available online at https://github.com/syriop-elisa/lead_time_bias.

## Appendix: Simulation study details

### Onset of breast cancer

The age of onset of breast cancer tumour was simulated based on breast cancer incidence rates (by 5-year age groups) in Sweden from 1973, which is the year before the introduction of mammography screening in Sweden and therefore our incidence rates are not influenced by screening. To convert incidence rates to probabilities of cancer onset, we assumed that all tumours were initiated 10 years prior to diagnosis. This assumption was only used to obtain probabilities of cancer onset. For each simulated dataset, the age at symptomatic detection, among individuals with breast cancer onset, was then simulated from a natural history model described in the following section (“Modelling tumour growth and symptomatic detection”). The available incidence rates were zero for ages younger than 20, so under our assumptions the earlier onset of disease was at age 10. The incidence rate values used for the simulation can be found at https://github.com/syriop-elisa/lead_time_bias.

### Modelling tumour growth and symptomatic detection

We simulated age and size of tumour at symptomatic diagnosis using a tumour growth model from onset of cancer that is described by Plevritis et al. [46] and by Abrahamsson and Humphreys [33].

We assume that each tumour is spherical and grows exponentially, with volume at time *t* years after onset equal to

8 $$\begin{aligned} V(t) = V_{0} \exp \left( \frac{t}{r} \right) \end{aligned}$$

where $V_0$ denotes the volume of the tumour when its diameter was 0.5 mm (unlikely to be detected at screening) and *r* is the inverse growth rate of the tumour. By defining ‘onset’ in this way, we assume that tumours are only detectable with (non-zero) probability from a volume corresponding to a diameter of 0.5 mm. Talkington and Durrett (2015) fitted different growth models to in vivo data on different cancers; for breast cancer data exponential growth fitted data better than a model based on Gompertz, logistic and power growth functions [47]. Different tumours grow at very different rates; to allow for this inverse growth rates were generated from a Gamma distribution with shape and rate parameters $\tau _1$ and $\tau _2$, respectively, and density function

9 $$\begin{aligned} f_R(r) = \frac{\tau _2^{\tau _1}}{\Gamma (\tau _1)} \exp (-\tau _2 r), \quad r \ge 0 \end{aligned}$$

The rate of symptomatic detection was assumed to be proportional to the volume of the tumour, so that

10 $$\begin{aligned} P(T_{\text {det}} \in [t, t + dt) | T_{\text {det}} > t) = \eta V(t) \hbox {d}t + o(dt) \end{aligned}$$

where $T_{\text {det}}$ is the time from onset to symptomatic detection.

Under Eqs. (8), (9) and (10), the conditional distribution of tumour volume at symptomatic detection, $V_{\text {det}}$, given a growth rate *r*, can be shown [46] to be

11 $$\begin{aligned} F_{V_{\text {det}} | R}(v) = 1 - \exp \left( -\eta r(v - V_{0})\right) , \quad v \ge V_{0}, \end{aligned}$$

and, using Eq. (8),

12 $$\begin{aligned} T_{\text {det}} = r \ln \left( \frac{V_{\text {det}}}{V_{0}} \right) \end{aligned}$$

Age at symptomatic detection was then calculated by adding the age at cancer onset to time $T_{\text {det}}$. For parameters $\tau _1$, $\tau _2$ and $\eta$ we choose values that are consistent with the tumour size distribution of breast cancers diagnosed during the years 1977–1979 in the Stockholm–Gotland region of Sweden as previously done in Andersson et al. [22]. Based on the marginal distribution of $V_{\text {det}}$ and assuming $\tau _1 = \tau _2$ for identifiability, (maximum likelihood) estimates were obtained and those were then used for the simulation. In specific, $\tau _1=\tau _2=1.385$ and $\eta =0.0002566$. We note that with these values the median tumour doubling time is 195 days which is close to the median tumour doubling time of 212 days reported by Fournier et al. [42] from a study measuring tumour size change in vivo, using sequential mammograms.

## Abbreviations

LLE: Loss in life expectancy
PLL: Proportion of life lost
FPM: Flexible parametric survival model
