# Supplementary material for: Assessing lead time bias due to mammography screening on estimates of loss in life expectancy
Source: Breast Cancer Res. 2022 Feb 23;24:15. doi: 10.1186/s13058-022-01505-3 (PMC8867879; doi:10.1186/s13058-022-01505-3)
Supplement: Supplementary file 1 — Additional file 1. Supplementary Tables S1, S2 and S3. [file 13058_2022_1505_MOESM1_ESM.pdf]

Additional file

Table S1: Proportion of screen detected cancers, among those with age of detection between 40-74 (when cancer screening is offered), across different simulation screening scenarios. All numbers are averages (with 2.5 and 97.5 percentiles in parenthesis) based on 200 simulations.

Attendance	Screening	Number diagnosed	% screen detected
Perfect	Low	2136 (2043 – 2221)	49.5 (47.5 – 51.7)
Perfect	Moderate	2196 (2098 – 2279)	62.2 (60.2 – 64.1)
Perfect	High	2262 (2163 – 2352)	71.8 (69.9 – 73.6)
Imperfect	Low	2106 (2016 – 2185)	38.4 (35.9 – 40.6)
Imperfect	Moderate	2154 (2052 – 2236)	49.3 (46.9 – 51.4)
Imperfect	High	2205 (2094 – 2300)	58.0 (56.0 – 60.0)

Table S2: Bias (absolute scale) for externally age-standardised 10-year relative survival (RS), loss in life expectancy (LLE) and proportion of life lost (PLL) across different screening sensitivities and attendance scenarios, with 95% confidence intervals based on the Monte Carlo error for bias (across 200 simulations). Bias was obtained as the difference to the setting in which no screening is imposed and all cases are symptomatic.

Attendance	Screening	10-Year RS	LLE	PLL
—	None (reference)	—	—	—
Perfect	Low	1.40 (1.19 – 1.60)	-0.28 (-0.31 – -0.25)	-1.19 (-1.35 – -1.03)
Perfect	Moderate	2.51 (2.31 – 2.71)	-0.45 (-0.48 – -0.42)	-1.95 (-2.11 – -1.79)
Perfect	High	3.85 (3.65 – 4.05)	-0.61 (-0.64 – -0.58)	-2.67 (-2.82 – -2.51)
Imperfect	Low	1.10 (0.90 – 1.29)	-0.22 (-0.25 – -0.19)	-0.92 (-1.07 – -0.76)
Imperfect	Moderate	1.87 (1.66 – 2.08)	-0.34 (-0.37 – -0.31)	-1.45 (-1.60 – -1.29)
Imperfect	High	2.87 (2.67 – 3.06)	-0.46 (-0.49 – -0.43)	-1.97 (-2.13 – -1.82)

Table S3: Average relative bias for externally age-standardised 10-year relative survival (RS), loss in life expectancy (LLE) and proportion of life lost (PLL) across different screening sensitivities and attendance scenarios, with 2.5 and 97.5 percentiles based on 200 simulations. The reference scenario is the setting in which no screening is imposed and all cases are symptomatic.

Attendance	Screening	10-Year RS	LLE	PLL
—	None (reference)	—	—	—
Perfect	Low	2.75 (0.49 – 5.22)	-3.47 (-5.69 – -1.28)	-2.69 (-5.11 – -0.43)
Perfect	Moderate	4.95 (1.63 – 8.13)	-5.58 (-8.78 – -2.33)	-4.42 (-7.47 – -1.22)
Perfect	High	7.58 (3.28 – 11.47)	-7.50 (-10.81 – -3.67)	-6.03 (-9.47 – -2.23)
Imperfect	Low	2.16 (0.20 – 4.60)	-2.67 (-5.08 – -0.88)	-2.07 (-4.49 – -0.33)
Imperfect	Moderate	3.68 (0.39 – 6.44)	-4.18 (-6.72 – -1.11)	-3.27 (-5.82 – -0.12)
Imperfect	High	5.65 (1.99 – 9.00)	-5.64 (-9.07 – -2.51)	-4.46 (-7.76 – -1.43)
